# Comparative Evaluation of the Prognostic Accuracy of IL-6 and Angiopoietin-2 for Early Severity Assessment in Acute Pancreatitis: A Systematic Review

**DOI:** 10.3390/diseases14010024

**Published:** 2026-01-07

**Authors:** Kairat Shakeev, Dmitriy Klyuyev, Alina Ogizbayeva, Aigul Baltabayeva, Olga Avdienko, Xenia Derevyashkina

**Affiliations:** 1 Department of Surgical Diseases, NCJC Karaganda Medical University, Karaganda 100000, Kazakhstan; 2 Institute of Life Sciences, NCJC Karaganda Medical University, Karaganda 100000, Kazakhstan; klyuev@qmu.kz; 3 Department of Emergency Medicine, Anesthesiology, and Resuscitation, NJSC Karaganda Medical University, Karaganda 100000, Kazakhstan; eleusizova.a@kgmu.kz; 4 Central Research Laboratory, NCJC Karaganda Medical University, Karaganda 100000, Kazakhstan; avdienko@qmu.kz; 5 Situation Center, Municipal State Enterprise on the Right of Economic Management Professor Kh. Zh. Makazhanov Multidisciplinary Hospital of the Health Department of the Karaganda Region, Karaganda 100000, Kazakhstan

**Keywords:** severe acute pancreatitis, pancreatic necrosis, interleukin-6, angiopoietin-2, APACHE II, biomarkers, persistent organ failure, multiorgan dysfunction syndrome

## Abstract

Background: Early identification of patients at risk for severe acute pancreatitis (SAP) remains a major clinical challenge. Circulating biomarkers reflecting systemic inflammation (IL-6) and endothelial dysfunction (Ang-2) have emerged as promising tools for improving early prediction of persistent organ failure and other adverse outcomes. Objective: To systematically synthesize and compare the diagnostic and prognostic performance of IL-6 and Ang-2 as early biomarkers of severity in adult patients with acute pancreatitis. Methods: This systematic review was conducted in accordance with PRISMA 2020 guidelines and prospectively registered in PROSPERO (CRD420251177279). PubMed, Scopus, and Web of Science were searched for studies published between 2000 and August 2025. Studies included adult patients (≥18 years) in whom IL-6 and/or Ang-2 levels were measured within 72 h of symptom onset or hospital admission, and where indices of diagnostic accuracy (AUC, sensitivity, specificity, or threshold values) were reported. Results: Fifteen cohort studies met the inclusion criteria. IL-6 demonstrated a consistent association with SAP and persistent organ failure, with AUC values ranging from 0.69 to 0.99; the highest accuracy was observed within the first 24 h. Specificity varied substantially across studies. Ang-2 showed uniformly high prognostic accuracy (AUC 0.79–0.98), reliably predicting persistent organ failure, multiorgan dysfunction, infected necrosis, and mortality. Conclusions: IL-6 exhibits high but heterogeneous diagnostic performance (AUC 0.69–0.99), whereas Ang-2 demonstrates consistently high accuracy (AUC 0.79–0.98) across study designs. Combined evaluation of inflammatory and endothelial pathways appears to offer the most robust strategy for early prediction of persistent organ failure in acute pancreatitis.

## 1. Introduction

Acute pancreatitis (AP) is a complex inflammatory disorder of the pancreas associated with substantial morbidity and mortality. Its global incidence continues to rise, reaching approximately 20–40 cases per 100,000 population, thereby imposing a considerable economic burden on healthcare systems. The clinical spectrum of AP ranges from mild interstitial edema to severe necrotizing disease [1,2,3,4]. The most consequential adverse outcome and the primary determinant of mortality is the development of severe acute pancreatitis (SAP). According to the revised Atlanta classification 2012, SAP is defined by the presence of persistent organ failure (POF) lasting 48 h or longer. While the overall mortality of AP is approximately 5%, mortality in SAP may reach 20–30% [2,3,5].

The pathophysiology of SAP is frequently initiated by the systemic inflammatory response syndrome (SIRS), often referred to as a “cytokine storm.” SIRS develops in nearly half of patients with AP and involves activation of both innate (neutrophils, monocytes, macrophages) and adaptive immune pathways. Ultimately, this process culminates in capillary leak syndrome, microvascular dysfunction, and failure of distant organs [6].

**Figure 1:**
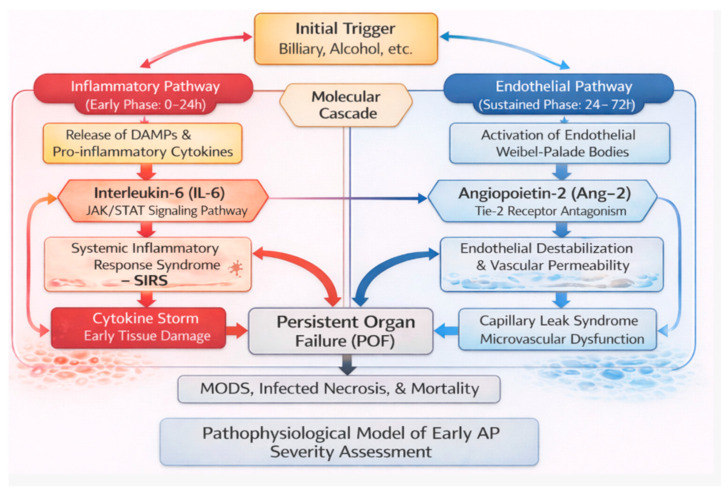
Pathophysiological model of early inflammatory and endothelial mechanisms in acute pancreatitis (IL-6 and Ang-2).

Early pathological stimuli induce acinar cell necrosis, leading to the release of damage-associated molecular patterns (DAMPs), including nucleotides and proteolytic enzymes, into the pancreatic interstitium. These signals promote recruitment and activation of pancreatic macrophages, resulting in the rapid production of pro-inflammatory cytokines, among which interleukin-6 (IL-6) plays a central role in amplifying systemic inflammatory responses. Elevated circulating IL-6 reflects the intensity of immune activation and has been associated with the development of persistent organ failure [7,8,9]. In parallel, inflammatory mediators induce endothelial activation and increased vascular permeability, processes in which angiopoietin-2 (Ang-2) acts as a key regulator of endothelial destabilization. The combined involvement of IL-6 and Ang-2 reflects inflammatory and endothelial processes that occur early during acute pancreatitis and are involved in the progression toward severe disease, providing a biological rationale for their evaluation as prognostic biomarkers in severe acute pancreatitis (Figure 1) [10].

Effective and accurate identification of patients at high risk for SAP is therefore essential for timely intensive management and appropriate triage. However, conventional prognostic systems—including APACHE II, the Ranson score, and BISAP—demonstrate suboptimal accuracy for early prediction of POF. Their main limitation lies in the fact that most scores rely on clinical and laboratory variables collected over 48–72 h and therefore reflect established organ dysfunction rather than early pathophysiological processes driving disease progression. In addition, tools such as APACHE II may be impractical in routine clinical settings due to the large number of variables required for calculation [11,12,13]. Consequently, biomarkers capable of capturing key pathophysiological mechanisms at the molecular level, before clinically apparent organ dysfunction emerges, are required for genuinely early prognostication.

Interleukin-6 (IL-6) is one of the most extensively studied early mediators of the inflammatory response. It is a multifunctional cytokine produced by T cells, macrophages, and endothelial cells, playing a central role in the systemic inflammatory response syndrome (SIRS) and acting as a principal regulator of acute-phase protein synthesis in hepatocytes. In parallel, IL-6 contributes to amplification of the systemic inflammatory response through activation of the JAK/STAT signaling pathway, thereby linking early cytokine release to downstream inflammatory signaling and tissue injury [14,15]. Serum IL-6 concentrations correlate closely with the severity of AP and precede the rise in other acute-phase reactants, such as C-reactive protein (CRP), thereby reflecting disease severity at the earliest stages [14,16]. IL-6 levels typically peak within the first 24 h after symptom onset, and multiple studies have demonstrated its statistically significant predictive value for complicated AP and SAP, with markedly higher concentrations observed in severe disease compared with non-severe forms (*p* < 0.001) [13,14]. Collectively, IL-6 reflects the inflammatory pathway underlying disease progression.

Angiopoietin-2 (Ang-2) is one of the most widely investigated biomarkers of endothelial dysfunction. It is an endothelial-derived factor that modulates the Tie-2 signaling pathway and promotes endothelial destabilization, increased vascular permeability, and impaired barrier integrity. Ang-2 concentrations rise rapidly during systemic inflammation and are strongly associated with the severity of critical illness [17,18]. Elevation of Ang-2 has been detected as early as the first hour after hospital presentation in patients with suspected infection, with levels proportional to disease severity and adverse outcomes (*p* < 0.0001). Among patients with unfavorable clinical courses, Ang-2 continues to increase, predicting the development of shock and mortality, while experimental data confirm its direct role in disrupting endothelial barrier function [19]. In SAP, Ang-2 levels are significantly higher than in non-severe AP and healthy individuals from the first day of illness, with sustained elevation beyond 48 h, distinguishing it from many other cytokines. Moreover, Ang-2 concentrations correlate with perfusion CT parameters, reflecting the extent of microvascular and endothelial dysfunction [20]. Altogether, Ang-2 reflects the endothelial pathway of AP pathogenesis.

Previous systematic reviews and meta-analyses, including those by Kumar et al. (2025) and Lv et al. (2021), have reported a high diagnostic accuracy of interleukin-6 (IL-6) and angiopoietin-2 (Ang-2) when evaluated as individual predictors of severe acute pancreatitis and organ failure [21,22]. These studies have provided valuable quantitative summaries of the available evidence; however, most analyses assessed each biomarker separately and were primarily based on pooled meta-analytic estimates, despite substantial heterogeneity in study design, patient populations, timing of biomarker assessment, and outcome definitions. In this context, the present review provides a structured qualitative synthesis of the available evidence on interleukin-6 and angiopoietin-2, incorporating the most recent data available up to August 2025, with the aim of establishing a comprehensive and biologically grounded framework for early risk stratification.

The aim of this systematic review is to critically synthesize and compare the available evidence on the diagnostic and prognostic value of IL-6 and angiopoietin-2 for early severity stratification and prediction of adverse outcomes, particularly persistent organ failure and mortality, in adult patients with acute pancreatitis.

## 2. Materials and Methods

This systematic review was conducted in accordance with PRISMA 2020 recommendations. A comprehensive literature search was performed in August 2025 across Web of Science, PubMed, and Scopus. The search strategy was developed a priori and is presented in Tables S1 and S2. The search was limited to original research articles published in peer-reviewed journals between 2000 and August 2025; studies published before 2000 were excluded because early investigations rarely reported key diagnostic accuracy metrics—such as AUC, sensitivity, specificity, or biomarker threshold values—required for prognostic synthesis and lacked standardized outcome definitions. Further restrictions included adult human populations (≥18 years) and English-language publications. Where available, database-specific filters were applied to include original research articles and to exclude reviews, editorials, conference abstracts, and animal studies. No restrictions were applied with respect to country, geographic region, or healthcare setting.

Inclusion criteria: Studies were considered eligible if they enrolled adult patients (≥18 years) with a diagnosis of acute pancreatitis based on the Atlanta classification; measured the relevant laboratory biomarkers within the first 72 h from symptom onset or hospital admission; and reported prognostic outcomes such as severe acute pancreatitis, persistent organ failure, infected necrosis, mortality, or the need for intervention. Only cohort observational studies or case–control studies published in English in peer-reviewed journals were included.

Exclusion Criteria: Exclusion criteria included studies conducted in non-human models; pediatric populations (<18 years); review articles, editorials, and conference abstracts; studies without early biomarker assessment (≥72 h); studies lacking extractable diagnostic accuracy metrics; and studies that did not define disease severity according to the revised Atlanta classification.

Titles and abstracts identified through the search were independently screened by two reviewers (ShKT and OAV). Full texts of potentially eligible studies were independently assessed by three reviewers (ADB, ShKT and OAV). Any disagreements regarding study inclusion or exclusion were resolved through discussion until full consensus was achieved. Screening was performed manually without the use of automation tools or machine-learning platforms.

### 2.1. Protocol Registration

This systematic review was prospectively registered in the International Prospective Register of Systematic Reviews (PROSPERO) under the title “Diagnostic and prognostic accuracy of serum Angiopoietin-2 and IL-6 for predicting disease severity in adult patients with acute pancreatitis: a systematic review” (Baltabayeva A., Shakeev K.; PROSPERO ID: CRD420251177279). The registration was formally approved on 14 November 2025.

The registered protocol included a detailed specification of the research question (PICO), predefined eligibility criteria, the complete search strategy for all databases, the outcome classification framework (harmonized severity categories A–D), and all data extraction fields. The protocol also stipulated that only adult patients (≥18 years) with acute pancreatitis were eligible; that biomarkers (IL-6 and Ang-2) must be measured during the early phase (preferably ≤72 h); and that studies were required to report prognostic performance metrics such as AUC, sensitivity, specificity, or biomarker thresholds. Any deviations from the protocol were minimal and are clearly described in the manuscript.

### 2.2. PROSPERO Reference

Aigul Baltabayeva, Kairat Shakeev. Diagnostic and prognostic accuracy of serum Ang-2 and IL-6 for predicting disease severity in adult patients with acute pancreatitis: a systematic review. PROSPERO 2025 CRD420251177279. Available at: https://www.crd.york.ac.uk/PROSPERO/view/CRD420251177279 (accessed on 1 December 2025).

### 2.3. Data Extraction

Data were independently extracted by two reviewers (BAD and ShKT) using a pre-designed and piloted data extraction form. The extraction template included key study characteristics (first author, year of publication, country, study design, clinical setting, sample size, participant demographics, timing of blood sampling, and inclusion criteria), biomarker-related information (analytical method, manufacturer, units of measurement, timing of assessment, and reported diagnostic/prognostic performance metrics), and outcome definitions. For each study, severity endpoints were mapped to the outcome classification system used in this review (classes A–D), including: persistent organ failure ≥48 h (class A), transient organ failure (class B), severity defined by scoring systems such as APACHE II, Ranson, or CTSI (class C), and hard clinical endpoints such as ICU admission, mortality, or infected pancreatic necrosis (class D).

For IL-6 and Ang-2, data extraction focused on early-phase measurements (preferably ≤72 h from symptom onset or hospital admission), reported threshold values, sensitivity, specificity, AUC, and measures of central tendency (means or medians) in severe versus non-severe disease groups. Studies reporting biomarker levels without prognostic accuracy metrics, those with unclear sampling time points, or those using inconsistent severity classifications were evaluated for eligibility and excluded when necessary.

Any discrepancies between the two reviewers were resolved through discussion, with a third reviewer (OAV) consulted when needed. Reference lists of all included studies were manually screened to identify additional relevant publications, which subsequently underwent the same screening and extraction procedures.

**Figure 2:**
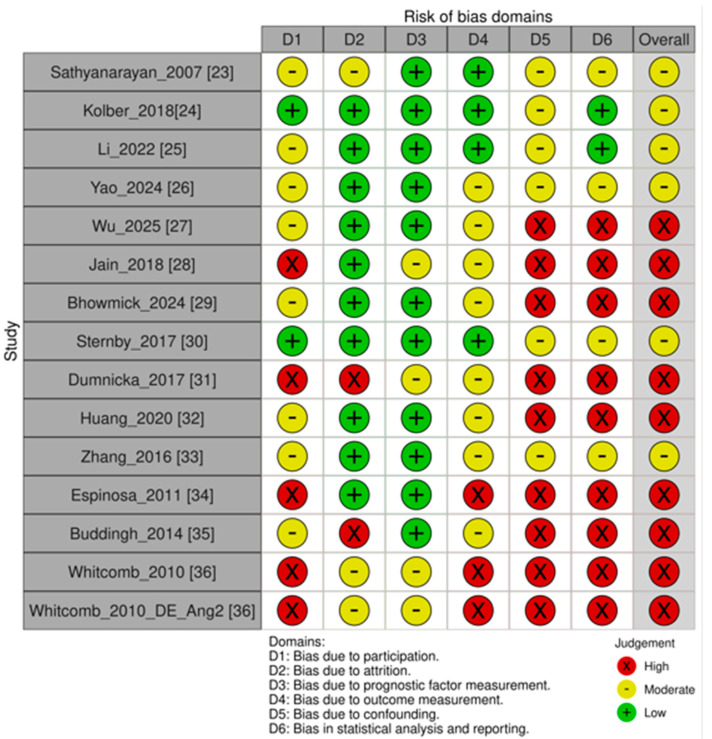
Risk of bias assessment of included studies using the QUIPS tool [23,24,25,26,27,28,29,30,31,32,33,34,35,36].

Quality assessment of the included studies was performed using the online tool available at https://mcguinlu.shinyapps.io/robvis/ (accessed on 1 December 2025), and the results are presented in Figure 2.

Risk of bias was evaluated using the QUIPS tool, which covers six domains: study participation, study attrition, prognostic factor measurement, outcome measurement, confounding control, and statistical analysis and reporting. The overall quality profile of the included studies demonstrated substantial methodological heterogeneity.

In most studies, the risk of bias in the domains of study participation and prognostic factor measurement was rated as low or moderate, reflecting adequate patient recruitment and the use of validated biomarker assessment methods. Outcome measurement was also predominantly assessed as low risk due to reliance on standardized clinical criteria, including the revised Atlanta classification and the Marshall score.

In contrast, the domains related to confounding control (D5) and statistical analysis (D6) were the most vulnerable. Ten out of fifteen studies demonstrated a high risk of bias because of the absence of multivariable regression, limited analytical modeling, or reliance on post hoc ROC analyses without internal validation. An additional four studies showed high risk in study participation or attrition due to incomplete data acquisition, retrospective design, or selection bias.

According to QUIPS guidance, the presence of at least one domain with high risk automatically results in a high overall risk classification. Consequently:

(1) 8 studies were classified as having a high overall risk of bias;
(2) 5 studies were classified as having a moderate overall risk;
(3) Only 2 studies were rated as having low or low-to-moderate risk.

Any discrepancies in domain assessments were resolved through consensus meetings involving all reviewers.

Given the considerable clinical heterogeneity (differences in study design, timing of blood sampling, and severity definitions) and methodological heterogeneity (high QUIPS risk), the decision was made not to perform a quantitative diagnostic meta-analysis. Instead, a narrative synthesis was applied, focusing on a critical appraisal of individual studies and an analysis of sources of heterogeneity.

### 2.4. Literature Search

The initial search across bibliographic databases identified 3477 records (PubMed: 1655; Scopus: 1612; Web of Science: 210). During the deduplication phase, 1058 records were removed, including system-identified duplicates (*n* = 949), automatically flagged irrelevant entries (*n* = 12), and publications excluded for technical reasons (*n* = 97). A total of 2442 unique records were retained for title and abstract screening, all of which were manually assessed. At this stage, 2406 records were excluded, primarily due to failure to meet the eligibility criteria: use of animal models (*n* = 372); non-relevant publication types such as reviews, meta-analyses or editorials (*n* = 1689); lack of original research content (*n* = 219); publications in languages other than English (*n* = 8); non-representative study designs (*n* = 116); and studies conducted in pediatric populations (*n* = 2).

Full-text assessment was performed for 36 studies, although full texts could not be obtained for five of them. Following detailed evaluation of the remaining 31 articles, 16 were excluded for the following reasons:

(1) Absence of required diagnostic or prognostic accuracy metrics (*n* = 11);
(2) Use of non-standard severity classification (*n* = 1);
(3) Absence of relevant clinical outcomes (*n* = 2);
(4) Non-representative study population (*n* = 1);
(5) Biomarker sampling outside the predefined diagnostic window of 72 h (*n* = 1).

**Figure 3:**
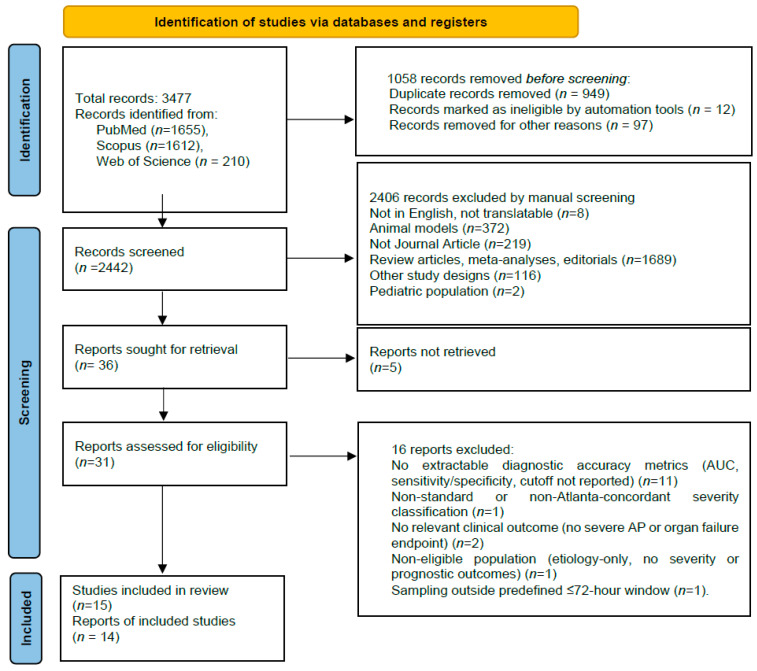
PRISMA 2020 flow diagram of study identification, screening, and inclusion.

In total, 15 studies met all inclusion criteria and were incorporated into the final systematic review. Manual screening of reference lists did not identify additional eligible publications. The study selection process is presented in Figure 3 [37].

Additional clarification is warranted regarding the publication by Whitcomb et al. (2010) [36], which includes two independent research centers: the United States cohort (UPMC, Pittsburgh) and the German cohort (Greifswald University). Although reported within a single article, these cohorts represent two distinct study populations with different patient characteristics, sample collection protocols and Ang-2 measurement time points. In this review, the findings from each center were treated as two separate studies because they meet the criteria for independent cohorts and provide individual diagnostic accuracy metrics.

For each included study, the extracted dataset also captured which biomarkers were measured (IL-6, Ang-2, or both). This allowed for both direct and indirect comparison of their prognostic performance in Section 3.3.

### 2.5. Characteristics of the Included Studies

The design and methodological features of the included studies are summarized in Table S3. Most investigations were conducted in China, India, and Poland, with single studies originating from Spain and Sweden. Additional studies were conducted in the Netherlands, as well as in the United States and Germany. In all studies, the diagnosis of acute pancreatitis was confirmed clinically and through laboratory testing, and in several reports further supported by abdominal imaging (ultrasound, CT or MRI), consistent with the prespecified inclusion criteria.

Across all included studies, comparable inclusion criteria were applied, reflecting early-phase acute pancreatitis. All studies enrolled adult patients aged 18 years or older experiencing a first episode of acute pancreatitis, with blood sampling performed during the early period of disease, typically within 24 to 48 h from symptom onset [23,24,25,26,27,28,29,30,31,32,33,34,35,36]. In some studies, the acceptable window extended to 72 h [23,35,36], and one study enrolled patients up to seven days from onset provided that a serum sample was available at admission [36].

Several investigations incorporated additional inclusion criteria, such as:

(1) Diagnosis confirmed through clinical, laboratory and imaging findings [24,29,30,31,32,33];
(2) Hypertriglyceridemic pancreatitis as the primary etiology [27];
(3) High predicted risk of severe disease (APACHE II ≥ 8, Imrie ≥ 3 or CRP > 150 mg/L) [35].

Exclusion criteria were generally similar across studies and aimed to eliminate conditions that could affect biomarker levels. The most common exclusions included:

(1) Chronic pancreatitis [24,27,28,31,33,36];
(2) Malignancies or suspicion of pancreaticobiliary tumors [24,29,31,32,35];
(3) Severe comorbid conditions (cardiopulmonary disorders, immunodeficiency, autoimmune disease) [25,26,27,29,32];
(4) Active infections or sepsis not related to pancreatitis [35];
(5) Post-ERCP pancreatitis or alcoholic etiology when the study focused on specific subgroups [27,35];
(6) Late presentation beyond 72 h, absence of baseline serum samples or incomplete clinical data [23,24,25,26,30,36];
(7) Refusal to participate or lack of informed consent [23,24,28,29,30,31,33,34].

Overall, the included studies enrolled patients in the early phase of acute pancreatitis while excluding chronic disease, severe comorbidities and scenarios in which biomarker levels might be non-representative or data incomplete. This ensured adequate comparability of the study cohorts and a consistent focus on early prognostic biomarkers IL-6 and Ang-2.

Most investigations were conducted as prospective observational cohort studies [23,24,28,30,31,32,33,36], providing standardized collection of biological samples and clinical data. Three studies employed retrospective cohort designs based on patient registries [26,27]. One study used a cross-sectional design [29], one followed a case–control approach [34], and one was a secondary analysis of a randomized clinical trial with prospective serum collection [35].

Significant variation was observed in the timing of blood sampling for IL-6 and Ang-2 measurement. In eight studies, samples were obtained within the first 24 h after symptom onset or hospital admission [24,29,30,31,32,33,36]. Two studies collected samples within the first 48 h [25,26], one within the first 6 h [27], and two within 72 h [23,28]. Two investigations used extended sampling schemes: serial serum collection during the first five days [35] or combined time points at 12 h and day 5 [34]. This distribution of sampling windows allowed assessment of both early inflammatory responses (IL-6) and later endothelial dysfunction (Ang-2).

In most studies, the severity of acute pancreatitis was defined according to the revised Atlanta classification (2012), which was applied in eleven investigations [24,25,26,27,28,29,30,31,32,33,35]. Ten studies defined severe disease as persistent organ failure lasting at least 48 h [24,25,26,28,29,30,31,33,36], and four additionally distinguished transient organ failure lasting less than 48 h [27,30,31,33]. Two studies did not specify the duration of organ dysfunction [32,35], while one defined severity based on a composite adverse clinical outcome [34].

The Modified Marshall score was used in ten studies [24,25,26,27,28,29,30,31,33,35], whereas two employed their own organ failure criteria [36]. Clinical severity indices were distributed as follows: APACHE II in nine studies [23,24,28,32,33,34,35,36], the Ranson score in seven [23,32,33,34,36], BISAP in two [24,31], the Glasgow/Imrie score in two [34,35], and the CTSI in two studies [27,33].

Clinical outcomes assessed across the included studies comprised severe acute pancreatitis (12 studies), persistent organ failure (10 studies), mortality (11 studies), admission to the intensive care unit (8 studies), infected pancreatic necrosis (4 studies) and multiorgan dysfunction syndrome (4 studies). One study evaluated an aggregated adverse clinical evolution [34].

Taken together, these data demonstrate a methodologically diverse yet broadly comparable evidence base focused on early biomarkers of systemic inflammation and endothelial dysfunction.

### 2.6. Patient Characteristics

Summary information on patient demographics is presented in Table S4. All 15 studies included adult patients with confirmed acute pancreatitis. Sample sizes varied considerably, ranging from 25 patients in the smallest cohort study [34] to 307 patients in the largest investigation [26], reflecting differences in study design and scale.

Patient age was reported heterogeneously, using either mean or median values. In most studies, age ranged from approximately 34–40 years in younger cohorts [23,28,29] to 60–72 years in studies enrolling older populations [30,31,34,35,36]. In two studies [25,33], demographic data were provided only for clinical subgroups, which precluded calculation of an aggregated cohort-wide estimate. Overall, the included studies encompassed a broad age spectrum, from young to elderly adults.

The proportion of male participants also varied substantially, from 36 percent in the study by Espinosa et al. [34] to 73 percent in Sathyanarayan et al. [23]. In most investigations, men constituted at least half of the study population, consistent with the known higher prevalence of acute pancreatitis among males. In one study [27], sex distribution was presented only by severity groups without reporting the overall proportion of male participants, and in another [33] demographic variables were similarly stratified by subgroups.

Despite differences in age and sex distribution, the included studies collectively represent a wide spectrum of clinical populations and provide sufficient demographic variability for evaluating the prognostic utility of IL-6 and Ang-2 in acute pancreatitis.

## 3. Results

### 3.1. IL-6: Summary of Key Results

IL-6 was investigated in eight studies [23,24,25,26,27,28,29,30]. The timing of blood sampling ranged from very early measurements obtained within the first 6 h after admission [27] to samples collected on day 3 after symptom onset [23,28]. Four studies obtained samples within the first 24 h after presentation or hospitalization [24,26,29,30], two within 48 h [25,26] and two within 72 h [23,28].

In most studies, IL-6 was measured using ELISA with commercial kits from Diaclone, R&D Systems or other manufacturers [23,25,28,29]. Kolber et al. used electrochemiluminescent immunoassay (ECLIA) on the Cobas platform [24], and Yao et al. applied multiplex microsphere-based immunofluorescence as part of a 12-cytokine panel [26]. The analytical method was not specified in Wu et al. [27]. All studies reported IL-6 concentrations in pg/mL.

Across all included studies, IL-6 was associated with severe acute pancreatitis, although its diagnostic accuracy varied considerably.

In Sathyanarayan et al., IL-6 measured on day 3, with a cut-off of 122 pg/mL, predicted organ failure with a sensitivity of 81.8 percent, specificity of 77.7 percent and an AUC of 0.823 [23]. In the multicenter study by Kolber et al., IL-6 concentrations measured at admission and on day 2 produced AUCs of 0.753 for severe pancreatitis, 0.767 for vital organ failure and 0.781 for ICU transfer or mortality, with corresponding cut-offs of 211–262 pg/mL, sensitivities of 57–62 percent and specificities of 82–88 percent [24].

In the retrospective Chinese study by Li et al., the AUC for IL-6 was 0.69 (95% CI 0.56–0.82) for severe pancreatitis, 0.72 for organ failure, 0.81 for infected pancreatic necrosis and 0.75 for mortality, with optimal cut-offs ranging from 54.16 to 219.3 pg/mL [25]. Yao et al. reported that a cut-off of 24.67 pg/mL within the first 48 h yielded a sensitivity of 87.2 percent, specificity of 66.9 percent and AUC of 0.79 (95% CI 0.73–0.85) for predicting severe disease [26].

The highest diagnostic accuracy was observed in the single-timepoint study by Bhowmick et al., where an IL-6 cut-off of 46.4 pg/mL within the first 24 h predicted severe pancreatitis with a sensitivity of 96.2 percent, specificity of 95.8 percent and an AUC of 0.99 (95% CI 0.91–1.00) [29]. In the ultra-early study by Wu et al. (sampling within 6 h), a cut-off of 27.4 pg/mL yielded a sensitivity of 87 percent and specificity of 73 percent, with an AUC of 0.86 [27]. In the prospective cohort by Jain et al., IL-6 levels above 160 pg/mL on day 3 were associated with severe disease (AUC 0.83, 95% CI 0.71–0.95; sensitivity 86 percent, specificity 82 percent) [28]. Sternby et al. reported that a cut-off of 50 pg/mL within 24–36 h discriminated mild from non-mild pancreatitis with an AUC of 0.72, sensitivity of 86 percent and specificity of 46 percent [30].

Overall, AUC values for IL-6 in predicting severe pancreatitis or persistent organ failure ranged from 0.69 to 0.99. Most studies reported high sensitivity with moderate specificity. Two investigations demonstrated additional prognostic relevance of IL-6 for infected pancreatic necrosis and mortality [25,29].

Several studies evaluated IL-6 as part of multivariable models. In the retrospective study by Wu et al., ultra-early IL-6 was identified as an independent predictor of adverse evolution; a combined model including IL-6, D-dimer and calcium improved outcome discrimination to an AUC of 0.88 [27]. In the prospective cohort by Jain et al., the presence of SIRS at admission together with IL-6 greater than 160 pg/mL on day 3 yielded a sensitivity of 79 percent, specificity of 95 percent, positive predictive value of 85 percent and negative predictive value of 93 percent for severe pancreatitis [28].

In the study by Li et al., IL-6 and C-reactive protein were included in regression models; IL-6 outperformed CRP in predicting infected necrosis and mortality but showed no clear advantage in predicting severe pancreatitis or organ failure [25]. In Kolber et al., IL-6 levels correlated moderately with Ang-2, procalcitonin, CRP and renal injury markers (KIM-1, L-FABP); IL-6 contributed to models assessing the risk of vital organ failure and ICU transfer [24]. Yao et al. analyzed IL-6 as part of multimarker panels incorporating cytokines and complications such as ANC, APFC, pleural effusion and ascites [26]. In the early study by Sathyanarayan et al., IL-6 measured on day 3 was significantly associated with organ failure, whereas TNF-alpha and IL-10 showed no prognostic value [23].

### 3.2. Ang-2: Summary of Key Results

Analysis of the eight studies included in this review demonstrated that Ang-2 is one of the most accurate and earliest biomarkers of severe acute pancreatitis. Despite methodological differences among studies, all reported consistent findings: elevated Ang-2 levels at admission or within the first 24 h were robustly associated with the development of persistent organ failure, severe local complications, infectious processes and mortality.

All studies measured Ang-2 using immunoassays, most commonly ELISA with commercial kits from R&D Systems. Sampling time points ranged from ultra-early measurements obtained within the first 12–24 h after symptom onset to repeated assessments at 48 h or day 5. This variability allowed evaluation of both early and dynamic changes in Ang-2 levels; however, even the first admission-time measurement consistently demonstrated significant prognostic value.

In the study by Dumnicka et al. [31], Ang-2 measured within the first 24 h showed high diagnostic accuracy for identifying severe pancreatitis (AUC 0.946), with a cut-off of 5.92 ng/mL yielding 100 percent sensitivity and 92 percent specificity. In the study by Huang et al. [32], Ang-2 also demonstrated strong accuracy for predicting severe gastrointestinal injury (AUC 0.916) and particularly for MODS, infected necrosis and mortality, with AUCs ranging from 0.90 to 0.98.

Zhang et al. [33] confirmed the diagnostic utility of Ang-2 for Atlanta-defined severity stratification, with AUC values ranging from 0.808 to 0.878 when distinguishing SAP from MAP. In the prospective study by Espinosa et al. [34], a cut-off of 10 ng/mL at 12 h after admission predicted adverse clinical evolution with an AUC of 0.97. Similar findings were reported in the multicenter study by Buddingh et al. [35], where Ang-2 demonstrated high accuracy for predicting multiorgan failure (AUC 0.784), infectious complications (AUC 0.816), intestinal ischemia (AUC 0.895) and mortality (AUC 0.865).

The studies by Whitcomb et al. [36] conducted in the United States and Germany also confirmed the high diagnostic value of Ang-2. In the U.S. cohort, the AUC reached 0.94, with a negative predictive value of 99 percent, indicating strong ability of low Ang-2 levels to exclude severe disease. In the German cohort, Ang-2 remained a significant marker (AUC 0.79) and outperformed CRP while demonstrating comparable prognostic accuracy to APACHE II.

Ang-2 was also an independent predictor of severe pancreatitis in multivariable models. In Zhang et al. [33], the odds ratio for SAP reached 12.1 at the optimal cut-off. In Buddingh et al. [35], higher Ang-2 levels were consistently associated with MOF, infectious complications and mortality, independently of other clinical variables. Even in studies with sampling limited to the early hours after admission, Ang-2 remained one of the most informative severity markers.

Overall, the evidence indicates that Ang-2 shows high predictive accuracy (AUC 0.79–0.98), applicable both to the early identification of severe pancreatitis and to forecasting complications requiring intensive care. When measured early, Ang-2 outperformed traditional markers, including CRP, and demonstrated prognostic accuracy comparable to or exceeding that of complex clinical scoring systems.

Collectively, the findings support Ang-2 as a reliable early biomarker of severe acute pancreatitis and a promising tool for clinical risk stratification in the first hours of disease onset.

### 3.3. Comparative Prognostic Performance of IL-6 and Angiopoietin-2

**Figure 4:**
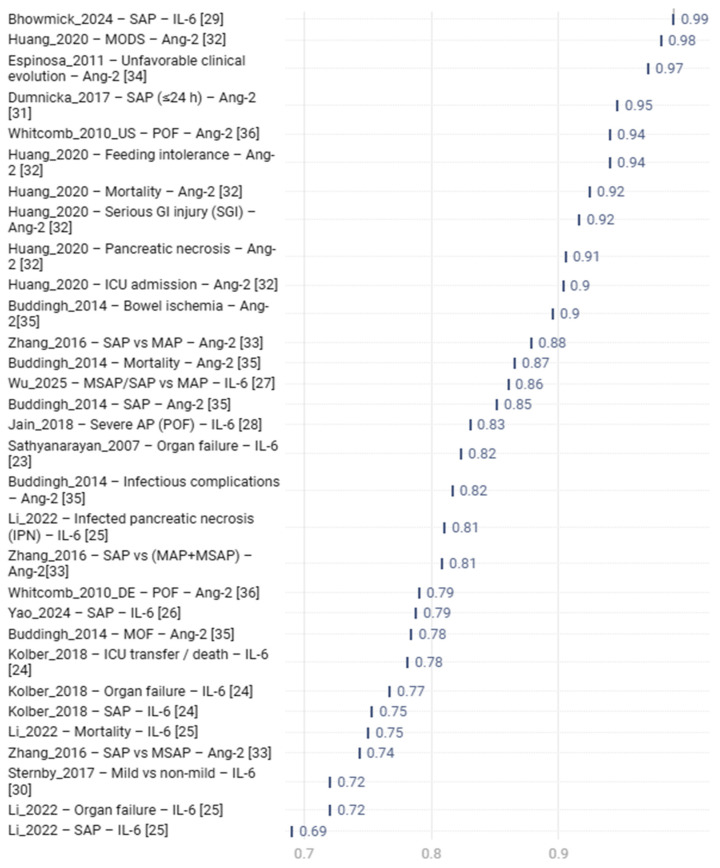
Prognostic AUC values of IL-6 and Ang-2 in acute pancreatitis.

Comparison of IL-6 and Ang-2 shows that the two biomarkers reflect distinct yet closely interconnected components of the early pathophysiology of acute pancreatitis (Figure 4) [38]. Despite heterogeneity in study designs, clinical endpoints and sampling times, their combined assessment reveals consistent patterns in diagnostic and prognostic accuracy.

Across studies, AUC values for IL-6 ranged from 0.69 to 0.99, forming a wide distribution primarily influenced by sampling time and the dynamic nature of the inflammatory response. The highest values were observed when IL-6 was measured in the ultra-early phase within the first hours or first day after symptom onset [26,27,29]. This pattern is evident on the plot, where Wu_2025, Yao_2024 and Bhowmick_2024 form the upper IL-6 cluster (AUC 0.86–0.99). In contrast, retrospective studies with later sampling showed moderate accuracy (AUC 0.69–0.75) [20]. This pattern corresponds to the kinetic profile of IL-6: concentrations rise sharply during early systemic inflammation and decline rapidly during stabilization.

Ang-2 demonstrated a more homogeneous performance profile. AUC values ranged from 0.79 to 0.98, with most clustering at 0.88 or higher [31,32,33,34,35,36]. This is reflected on the diagram: studies by Huang_2020, Espinosa_2011, Buddingh_2014 and both Whitcomb_2010 cohorts form a dense block of high AUC values, highlighting the stability of Ang-2 as a marker of endothelial dysfunction. The highest values were associated with outcomes linked to microvascular injury, including MODS, intestinal ischemia, infected necrosis and mortality.

Only two studies evaluated IL-6 and Ang-2 in the same cohort, enabling direct comparison. In the Whitcomb_2010 UPMC cohort, both biomarkers showed high accuracy for predicting persistent organ failure, with IL-6 having a slight numerical advantage (AUC 0.950 vs. 0.94) [36]. Kolber_2018 did not report comparative ROC curves but documented a positive correlation between IL-6 and Ang-2, especially on day 2 (R = 0.54, *p* = 0.004) [24], indicating joint activation of inflammatory and endothelial pathways.

Indirect comparison across all studies revealed a consistent pattern: Ang-2 demonstrated more stable prognostic accuracy across severe outcomes, while IL-6 captured the early inflammatory surge but was more sensitive to timing and clinical variability. On the plot, IL-6 spans the full range from moderate to near-perfect accuracy, while Ang-2 is concentrated in the consistently high zone.

These differences align with the biological roles of the biomarkers. IL-6 reflects early systemic inflammation and is closely associated with SIRS, making it a sensitive but less specific indicator of severe complications. Ang-2 reflects endothelial injury, vascular permeability and microcirculatory impairment, processes that underlie persistent organ failure. Accordingly, Ang-2 shows the highest AUC values for outcomes where endothelial dysfunction plays a central role.

In summary, both IL-6 and Ang-2 have significant prognostic value but reflect different stages of disease evolution. IL-6 provides a sensitive measure of early inflammatory burden, while Ang-2 offers a stable indicator of endothelial injury and risk of severe complications. The most accurate prediction of severe acute pancreatitis is achieved when IL-6 and Ang-2 are used in combination, capturing both systemic inflammation and endothelial dysfunction. This dual-biomarker approach offers a more comprehensive understanding of early disease progression and may support integrated risk-stratification algorithms in acute pancreatitis.

## 4. Discussion

The findings of this systematic review demonstrate that Ang-2 and IL-6 represent two fundamental and complementary components of the early pathophysiology of acute pancreatitis, offering valuable opportunities for risk stratification within the first hours and days of disease onset. Despite substantial methodological heterogeneity across studies—including variation in sampling times, analytical techniques and definitions of severity—the collective evidence indicates strong prognostic potential for both biomarkers, with distinct and mutually reinforcing clinical roles. To facilitate comparison of their biological behavior, temporal profiles, and prognostic performance, the key findings for interleukin-6 and angiopoietin-2 are summarized in Table S5.

IL-6 consistently emerges as a marker of the early systemic inflammatory response. Its concentration increases within hours of acute inflammation and directly reflects the intensity of the SIRS-driven phase of the disease. Across diverse populations and sampling windows, IL-6 demonstrated the ability to discriminate between severe and non-severe acute pancreatitis with moderately high accuracy [23,24,25,26,27,28,29,30]. Reported AUC values ranged from 0.69 [25] to 0.99 [29], a variability attributable to the dynamic nature of IL-6 and differences in study design. Despite this variation, IL-6 showed a robust association with the development of organ failure, and in some studies, it outperformed CRP for predicting infected necrosis and mortality [25,28]. IL-6 also enhanced the prognostic accuracy of clinical scores such as SIRS, indicating its role as an integrative marker linking inflammatory and clinical components of early disease progression [28]

In contrast, Ang-2 is a marker of endothelial dysfunction-the pathway underlying vascular leakage, hypovolemia, microcirculatory impairment and the development of persistent organ failure. Evidence from multiple independent cohorts demonstrates that Ang-2 has more stable prognostic performance than IL-6, particularly for predicting persistent organ failure, MODS, infected necrosis, intestinal ischemia and mortality [31,32,33,34,35,36]. Reported AUC values for Ang-2 clustered predominantly between 0.85 and 0.98 [31,32,33,34,35], with the highest values observed for outcomes directly associated with endothelial injury [32,33,34,36].

Importantly, Ang-2 maintained high prognostic accuracy regardless of sampling time, both at admission and within the first 48 h of disease onset [31,32,33,34,36]. Its ability to predict MODS, infected necrosis and ICU requirement across studies from Europe, Asia and the United States underscores its biological consistency [31,32,33,34,36]. In addition, Ang-2 remained elevated throughout the first week in patients with ongoing organ dysfunction, making it suitable for dynamic monitoring, unlike IL-6, which typically declines more rapidly [36].

The most analytically informative data come from the few studies that measured IL-6 and Ang-2 in the same cohort. Only two such head-to-head comparisons were available, underscoring a notable gap in the literature. In the Whitcomb (UPMC, USA) cohort, both biomarkers were measured simultaneously: IL-6 achieved an AUC of 0.95 for predicting persistent organ failure, while Ang-2 reached 0.94. Although numerically similar, IL-6 showed a rapid decline after its peak, whereas Ang-2 remained elevated for several days in patients with persistent organ dysfunction [36]. In the second study by Kolber et al. [24], Ang-2 demonstrated higher AUC values for most endpoints, including severe pancreatitis, ICU requirement and clinically significant organ failure. Correlation between IL-6 and Ang-2 was moderate (R = 0.54 on day 2), increasing over time, consistent with their involvement in interconnected but distinct pathogenic pathways.

Taken together, these head-to-head data support an important conclusion: IL-6 is more informative during the ultra-early phase of disease [24,36], whereas Ang-2 provides superior accuracy and clinical relevance for predicting persistent organ failure and endothelial dysfunction–related complications during the subsequent phase [31,32,33,34,35,36]. This complementarity provides a compelling rationale for combined biomarker use.

Interpretation of these findings, however, must consider the heterogeneity of included studies. Three major factors exert the greatest influence: sampling time, severity classification and analytical platform [23,24,25,26,27,28,29,30,31,32,33,34,35,36]. IL-6 exhibits rapid biological fluctuations; measurements at different time points (6 h, 24 h, 48 h, 72 h or day 3) produce differing thresholds and diagnostic performance [23,24,25,26,27,28,29,30]. Ang-2 is less sensitive to sampling time but is reported in multiple units (pg/mL, ng/mL), complicating data aggregation [31,32,33,34,35,36]. Variability between the 1992 and 2012 Atlanta classifications also affects the frequency of severe outcomes and ROC modeling.

Nevertheless, consistent patterns emerge across studies. Both biomarkers are clinically meaningful but reflect different aspects of disease evolution and therefore fulfill distinct diagnostic functions. IL-6 signals initiation of a strong inflammatory response [23,24,25,26,27,28,29,30]. Ang-2 reflects structural and functional endothelial injury, the downstream consequence of this inflammation [31,32,33,34,35,36].

These findings support a dual-marker clinical model: IL-6 may serve as an early screening tool within the first hours after admission [23,24,25,26,27,28,29,30], identifying patients who require intensified monitoring or early supportive care. Ang-2, measured at admission or within the first 24 h, can provide a more accurate assessment of risk for persistent organ failure and complications [31,32,33,34,35,36], including infectious processes and intestinal ischemia. Thus, an integrated IL-6 + Ang-2 panel represents a promising approach that may outperform existing clinical scores and single biomarkers.

Despite clear advantages, the incorporation of Ang-2 and IL-6 into routine clinical practice requires further research. Multicenter prospective studies with standardized sampling times, unified analytical platforms and harmonized outcome definitions are needed [23,24,25,26,27,28,29,30,31,32,33,34,35,36]. Moreover, the distinct temporal profiles of IL-6 and Ang-2 highlight the need to explore biomarker dynamics, which may inform the development of multi-stage prognostic algorithms. The issue of optimal cutoff values also remains unresolved, as thresholds vary substantially across studies and populations.

Overall, this systematic review confirms that IL-6 and Ang-2 are highly informative early biomarkers of severe acute pancreatitis. Their combined use may significantly improve early risk stratification, optimize patient triage and increase treatment effectiveness-particularly within the first 24–72 h, when clinical decisions exert the greatest influence on outcomes.

### Limitations

This systematic review has several limitations.

First, heterogeneity of the available data remains a limitation for the development of uniform clinical recommendations for IL-6 and angiopoietin-2 in acute pancreatitis. Reported IL-6 cut-off values vary widely across studies, ranging from 24.67 pg/mL to 262 pg/mL, largely due to differences in disease severity and blood sampling time. For angiopoietin-2, direct comparison is further limited by inconsistent reporting units and assay platforms, with concentrations expressed in pg/mL, ng/mL, μg/L, or mg/L across studies. Timing of sample collection also differs substantially, from 6 to 72 h after symptom onset, which is particularly relevant for IL-6 given its rapid early peak and subsequent decline. Additional heterogeneity arises from methodological differences, including the use of different Atlanta classification versions (1992 vs. 2012) and organ failure assessment tools (Marshall vs. SOFA). Together, these factors preclude the definition of universal biomarker thresholds and limit the feasibility of quantitative meta-analysis. Standardization of analytical methods, reporting units, sampling time points, and outcome definitions is therefore required before IL-6 and angiopoietin-2 can be reliably integrated into clinical practice.

Second, most studies were single-center with relatively small sample sizes, and eight of the fifteen studies were rated as having a high overall risk of bias according to the QUIPS tool, thereby limiting the reliability of aggregated conclusions.

Third, only English-language publications were included, which may have introduced language and publication bias.

Fourth, the costs associated with IL-6 and angiopoietin-2 testing were not systematically reported across the included studies, limiting conclusions regarding their economic feasibility in routine clinical practice.

Finally, direct head-to-head comparisons of IL-6 and Ang-2 were available only in a small subset of studies, restricting the strength of conclusions regarding their relative prognostic performance.

## 5. Conclusions

This systematic review demonstrates that both IL-6 and Ang-2 have meaningful diagnostic and prognostic value in the early risk stratification of adults with acute pancreatitis. IL-6 consistently reflects the intensity of the early systemic inflammatory response and enables identification of patients at risk for adverse outcomes within the first hours and days of disease onset. However, its specificity and temporal stability are limited by pronounced biological and methodological variability.

Ang-2 shows higher and more consistent prognostic accuracy, particularly for persistent organ failure, MODS, infected necrosis, intestinal ischemia, need for intensive care and mortality. In all available head-to-head studies, Ang-2 performed at least as well as IL-6 and frequently exceeded it in terms of AUC, sensitivity and specificity, underscoring its central role as a marker of endothelial dysfunction and microvascular injury.

Taken together, IL-6 and Ang-2 should not be viewed as competing markers but as complementary indicators that capture sequential aspects of the pathophysiology of severe acute pancreatitis. Combined use of these biomarkers within standardized protocols has the potential to substantially improve early prognostic accuracy and patient triage. Implementation into routine practice will require large prospective multicenter studies with harmonized outcome definitions, standardized analytical platforms and external validation of proposed thresholds and combined predictive models.

## Supplementary Materials

The following supporting information can be downloaded at: https://www.mdpi.com/article/10.3390/diseases14010024/s1, Tables S1 and S2: Search strategies and terms used across databases, PICO framework defining the review question. Table S3: Study and patient characteristics of the included studies. Table S4: Biomarker measurement characteristics and diagnostic performance in included studies. Table S5: Summary of Key Findings for Interleukin-6 and Angiopoietin-2 in Acute Pancreatitis.

## Abbreviations

The following abbreviations are used in this manuscript:

AP	Acute Pancreatitis
SAP	Severe Acute Pancreatitis
MSAP	Moderately Severe Acute Pancreatitis
IL-6	Interleukin-6
Ang-2	Angiopoietin-2
CRP	C-Reactive Protein
OF	Organ Failure
POF	Persistent Organ Failure
IPN	Infected Pancreatic Necrosis
MODS	Multiple Organ Dysfunction Syndrome
ICU	Intensive Care Unit
AUC	Area Under the Curve
CTSI	Computed Tomography Severity Index
SIRS	Systemic Inflammatory Response Syndrome
ELISA	Enzyme-Linked Immunosorbent Assay
APACHE II	Acute Physiology and Chronic Health Evaluation II
