# Supplementary material for: Comparative Evaluation of the Prognostic Accuracy of IL-6 and Angiopoietin-2 for Early Severity Assessment in Acute Pancreatitis: A Systematic Review
Source: Diseases. 2026 Jan 7;14(1):24. doi: 10.3390/diseases14010024 (PMC12839801; doi:10.3390/diseases14010024)
Supplement: Supplementary file 1 [file diseases-14-00024-s001.zip › Table S1,2.pdf]

Table S1. Search strategies and terms used across databases.

Database		Search Terms
Web of Science	#1	TS=("Interleukin-6" OR "IL-6" OR "Interleukin 6" OR "Angiopoietin-2" OR "Ang-2" OR "Ang2" OR "Angiopoietin 2" OR "Endothelial Biomarkers" OR "Cytokine*" OR "Inflammatory Biomarker*" OR "Vascular Permeability Marker*")
	#2	TS=("Acute Pancreatitis" OR "Severe Acute Pancreatitis" OR "SAP" OR "Moderately Severe Acute Pancreatitis" OR "MSAP" OR "Pancreatic Necrosis" OR "Necrotizing Pancreatitis" OR "Organ Failure" OR "Persistent Organ Failure" OR "POF" OR "Systemic Inflammatory Response")
	#3	TS=("Prognos*" OR "Predict*" OR "Prediction" OR "Diagnostic Accuracy" OR "ROC" OR "Receiver Operating Characteristic" OR "Sensitivity" OR "Specificity" OR "AUC" OR "Cutoff" OR "Biomarker Performance" OR "Early Phase" OR "Early Severity")
	#4	#1 AND #2 AND #3
PubMed	#1	(“Interleukin-6”[Mesh] OR “Interleukin-6/blood”[Mesh] OR “IL-6”[Title/Abstract] OR “Angiopoietin-2”[Mesh] OR “Angiopoietin-2/blood”[Mesh] OR “Ang-2”[Title/Abstract] OR “Biomarkers”[Mesh] OR “Cytokines”[Mesh] OR “Endothelial Cells/metabolism”[Mesh])
	#2	(“Pancreatitis”[Mesh] OR “Pancreatitis, Acute Necrotizing”[Mesh] OR “Severe Acute Pancreatitis”[Title/Abstract] OR “Acute Pancreatitis”[Title/Abstract] OR “Pancreatic Necrosis”[Title/Abstract] OR “Systemic Inflammatory Response Syndrome”[Mesh] OR organ failure[Title/Abstract] OR “persistent organ failure”)
	#3	(“Severity of Illness Index”[Mesh] OR “Prognosis”[Mesh] OR “ROC Curve”[Mesh] OR “Area Under Curve”[Mesh] OR “Sensitivity and Specificity”[Mesh] OR prediction[Title/Abstract] OR prognos*[Title/Abstract] OR cutoff[Title/Abstract] OR AUC[Title/Abstract])
	#4	#1 AND #2 AND #3
Scopus	#1	(Interleukin-6 OR IL-6* OR “Interleukin 6”* OR Angiopoietin-2* OR Ang-2* OR Biomarker* OR Cytokine*)<br>AND<br>(Acute Pancreatitis* OR “Severe Acute Pancreatitis”* OR SAP* OR MSAP* OR “Pancreatic Necrosis”* OR “Necrotizing Pancreatitis”* OR “Organ Failure”* OR “Persistent Organ Failure”)<br>AND<br>(Prognos OR Predict* OR Severity* OR “Diagnostic Accuracy”* OR ROC* OR “Receiver Operating Characteristic”* OR AUC* OR Sensitivity* OR Specificity* OR Cutoff*)*

Table S2. PICO framework defining the review question.

Components of PICO	Definition
Population	Adults (≥18 years) with acute pancreatitis confirmed by clinical, laboratory, and/or imaging criteria; blood sampling performed during the early phase (preferably ≤72 hours from symptom onset or hospital admission).
Intervention	Interleukin-6 (IL-6) measured at baseline for the purpose of early prediction of disease severity.
Comparison	Angiopoietin-2 (ANG-2) measured at the same time point and within the same cohort (head-to-head comparison prioritized); if unavailable, indirect comparison through pooled narrative synthesis.
Outcome	Primary outcome: severe acute pancreatitis (harmonized severe), with mapping of study-specific definitions into unified outcome classes:<br><br>Class A: Revised Atlanta 2012 persistent organ failure (≥48 h) based on modified Marshall/SOFA;<br>Class B: organ failure without confirmation of persistence;<br>Class C: “severity by scoring systems” (APACHE, Ranson, CTSI; threshold specified);<br>Class D: hard clinical endpoints used as severity proxies (ICU admission, mortality, infected pancreatic necrosis).<br>Sensitivity analysis: restricted to Class A outcomes (POF ≥48 h).
