# Supplementary material for: Comparative Evaluation of the Prognostic Accuracy of IL-6 and Angiopoietin-2 for Early Severity Assessment in Acute Pancreatitis: A Systematic Review
Source: Diseases. 2026 Jan 7;14(1):24. doi: 10.3390/diseases14010024 (PMC12839801; doi:10.3390/diseases14010024)
Supplement: Supplementary file 1 [file diseases-14-00024-s001.zip › Table S3.pdf]

Table 3. Study and patient characteristics of the included studies.

Study_ID	Year	Country	Design	N_total	Age_Mean_SD	Male %	Inclusion_Criteria_Short	Exclusion_Criteria_Short	Sampling_Window_h	Outcome_Definition_FreeText	Outcome_Term	Organ_Failure_Score (Marshall/SOFA/Other)	OF_Persistence (>=48h/<48h/unclear/NA)	Severity_Score_Name (APACHE/Ranson/CTSI/Other)	Severity_Score_Threshold	Hard_Outcome_Type (ICU/Mortality/IPN)	Time_to_Outcome_Assessment (hours/days)	Outcome_Unit_Details	Notes_Outcome
Sathyanarayan_2007 [23]	2007	India	prospective observational	108 (total); 30 for cytokine subset	40.3 ± 13.9	73.3	Adults with acute pancreatitis presenting ≤72 h after onset	Presentation >72 h, incomplete data, refusal of consent	≤72	Atlanta 1992: severe AP defined as ≥1 organ failure and/or APACHE II ≥8	SAP / organ failure	NA	any duration	APACHE II	≥8	NA	up to 14 days follow-up	Organ failure, severe disease	Organ failure any duration per Atlanta 1992 (used for harmonized class B).
Kolber_2018 [24]	2018	Poland	prospective observational	95	48 ± 16.5	68	Adults ≥18 years with AP admitted ≤24 h from symptom onset	Symptoms > 24 h, chronic pancreatitis, pancreatic cancer, chronic liver disease, neoplasia, no consent	≤ 24	Revised Atlanta 2012: SAP defined as POF (≥48 h) in Marshall score ≥ 2 (cardiovascular, respiratory, renal)	SAP / vital organ failure	Marshall	≥ 48 h (persistent)	Ranson, BISAP, BALI	Ranson ≥ 3, BISAP ≥ 3, BALI ≥ 3 points	ICU transfer or death (analyzed separately)	first 48 h for prediction; in-hospital follow-up to discharge	POF ≥ 48 h or SAP per 2012 Atlanta	Primary outcomes included SAP, vital organ failure, and ICU transfer/death; SAP prevalence 8.5%.
Li_2022 [25]	2022	China	Retrospective cohort (from prospectively maintained database)	67	Mean ≈ 48 years (SD not reported; median/IQR available)	29.9	Adults admitted ≤ 48 h after symptom onset; IL-6 and CRP measured on admission	Missing IL-6/CRP data; admission > 48 h; other specific reasons in Figure 1	≤ 48	SAP = POF ≥ 48 h based on Modified Marshall Score ≥ 2 in any system (Revised Atlanta 2012)	SAP (SAP) / Organ failure / Infected pancreatic necrosis (IPN) / Mortality	Marshall	≥ 48 h	NA	NA	ICU admission / Mortality / IPN (analyzed separately)	In-hospital (follow-up until discharge)	Organ failure and other severe outcomes assessed per Revised Atlanta 2012	Cohort results: organ failure 68.7 %; persistent OF 49.3 %; SAP 52.2 %; IPN 17.9 %; mortality 9 %.
Yao_2024 [26]	2024	China	Retrospective study	307	NA (median 45 [IQR 34–63])	46.9	First diagnosed AP; age 18–80; 12 cytokines tested within 48 h of symptom onset; chest and abdominal CT	Chronic pancreatitis; long-term immunomodulatory therapy; severe cardiovascular/pulmonary disease, AIDS, autoimmune disease, malignancy; pregnancy; incomplete data	≤ 48	SAP defined as persistent organ dysfunction (>48 h) based on the modified Marshall scoring system (score ≥ 2 in any system)	SAP (SAP)	Marshall	≥ 48 h	NA	NA	ICU admission; Death (reported)	In-hospital	Early in-hospital complications assessed: ANC, APFC, pleural effusion, ascites	SAP n=47 (15.3%); organ failure n=69 (22.5%); ICU admission n=25 (8.1%); death n=1 (0.3%).
Wu_2025 [27]	2025	China	Retrospective study	110	Reported by groups (e.g., 44.3 ± 4.1 vs 33.6 ± 4.9 years)	Not explicitly stated as a single percentage (sex distrib	HTGP; TG ≥ 11.3 mmol/L at onset (or 5.65–11.3 mmol/L if other causes excluded); enhanced CT within 72 h of admission	Alcoholic AP; post-ERCP pancreatitis; chronic pancreatitis; chronic renal dysfunction	≤ 6 (from admission)	MSAP: transient organ failure (recovered within 48 h) and local/systemic complications; SAP: POF (> 48 h) with modified Marshall score ≥ 2 (Revised Atlanta 2012)	MSAP/SAP severity (vs MAP)	Modified Marshall score	Mixed (MSAP transient; SAP persistent)	NA	NA	Complications reported; organ failure, SIRS, infectious complications (percentages provided)	In-hospital (final clinical outcomes)	Grouped as MAP (n=56) vs MSAP/SAP (n=54)	Severity categorization per 2012 Revised Atlanta; enhanced CT within 72 h of admission.

						ution compared; P > 0.05)													
Jain_2018 [28]	2018	India	Prospective single-center observational cohort	115	39.4 ± 14.6 years	57 %	First episode of acute pancreatitis; hospitalization within 7 days of onset	Chronic pancreatitis; recurrent acute pancreatitis; refusal of consent	≤ 72 (cytokine cohort measured at day 3 of onset)	Severe AP defined as POF (>48 h) with Modified Marshall score ≥ 2 (Revised Atlanta 2012)	SAP (POF)	Modified Marshall	≥ 48 h	NA	NA	Mortality (20/115); Infected pancreatic necrosis (26/115)	In-hospital; early POF as primary outcome	Severe AP 37/115 (32%); Moderately severe 25/115 (22%); Mild 53/115 (46%)	Persistent SIRS and IL-6 were evaluated as predictors of severe AP.
Bhowmick_2024 [29]	2024	India	Cross-sectional study	50	34.34 ± 11 years	70 % (35/50)	Diagnosed acute pancreatitis presenting within 48 h of pain onset	Immune-deficient condition; severe cardiac disease; preexisting hepatic disorder; preexisting renal compromise	Blood drawn within 24 h of admission	SAP defined as POF >48 h per Revised Atlanta 2012; organ failure by Modified Marshall score	SAP (SAP) vs MSAP vs MAP	Modified Marshall	(≥48h/<48h/unclear/NA) - ≥48 h (SAP)	NA	NA	NA	Modified Marshall assessed on day 0 and day 3 of hospitalization	Classification into MAP/MSAP/SAP according to RA2012 and Modified Marshall scoring	ROC used to derive cutoffs for IL-6, IL-8, TNF-α to predict SAP; pair-wise AUC comparisons performed.
Sternby_2017 [30]	2017	Sweden	Prospective observational cohort	175	Median 66 (range 19–97)	~53 % ("just over half were male")	Adults ≥18 y with acute pancreatitis diagnosed by ≥2/3 criteria (clinical, labs, imaging)	Insufficient Swedish language comprehension; missing blood samples	Within first 24–36 h (early course)	Revised Atlanta Classification 2012: • Moderately severe AP = transient organ failure (<48 h) and/or local/systemic complications • Severe AP = POF (≥48 h)	SAP (moderately severe + severe)	Marshall score	**both** (transient <48h & persistent ≥48h	NA	NA	NA	Up to discharge or 30-day clinical course	Non-mild AP (moderately severe + severe), defined per Atlanta 2012 criteria using Marshall organ failure score	Non-mild = moderate + severe per Revised Atlanta 2012; transient and persistent OF considered separately but combined for ROC analysis.
Dumnicka_2017 [31]	2017	Poland	Prospective observational study	69 AP patients; 21 healthy controls	69 ± 18 years (overall)	50.7 % (35/69)	Admission within 24 h from onset; adults with AP; informed consent	Coagulopathies/anticoagulant therapy; chronic pancreatitis; neoplasms; chronic liver diseases (cirrhosis/viral hepatitis)	24 h and 48 h from onset (on admission and Day 2)	SAP = POF (>48 h); MSAP = transient OF (<48 h) and/or local/systemic complications; MAP = no OF/complications (RA2012).	SAP vs MSAP vs MAP	Modified Marshall (per RA2012)	≥48 h (SAP); <48 h (MSAP)	BISAP; Glasgow (reported as baseline indices)	NA	Mortality reported (higher in DIC subgroup)	Admission (≤24 h) and Day 2 (48 h) assessments; severity classified over hospital stay	ROC AUCs for predicting SAP and MSAP+SAP using Ang-2, sFlt-1, and hemostasis tests.	Ang-2 best for SAP at admission (AUC 0.946)
Huang_2020 [32]	2020	China	Prospective observational study	270 (AP patients n=170; healthy controls n=100)	55.4 ± 17.2 years (AP patients)	69.4 % (118/170) in AP pati	Admission within 24 h after onset of AP; adults; diagnosis per criteria above	Serious systemic diseases; malignancy; active autoimmune disease; serious	Blood collected on admission	AGI graded 1–4 per ESICM recommendations; SGI defined as AGI grades 3–4. MODS evaluated by Marshall MODS score; pancreatic necrosis by imaging; feeding	Serious gastrointestinal injury (SGI: AGI grade 3–4) vs non-SGI (AGI grade 1–2); adverse outcomes (MODS,	Marshall MODS score (organ score >2 denotes	Unclear (persistence not specified)	APACHE II; Ranson (reported as comparators)	NA	ICU admission; mortality; MODS; pancreatic necrosis (all analyzed)	During index hospitalization (prospective follow-up)	ROC analysis performed for SGI (AGI 3–4) and for adverse outcomes (MODS, necrosis, ICU, feeding intolerance, mortality)	Ang-2 increased across AGI grades 1→4; conventional tools (Ranson, APACHE II, CRP) did not discriminate AGI grades effectively.

						ents		infectious disease	within 24 h after onset (single timepoint)	intolerance = TPN >1 week.	pancreatic necrosis, ICU admission, feeding intolerance, mortality)	dysfunction/failure)							
Zhang_2016 [33]	2016	China	Prospective cohort (serum collected on admission)	120 (MAP 40; MSAP 43; SAP 37)	NA (group-wise values reported)	NA (per subgroup: MAP 57.5%; MSAP 67.4%; SAP 59.5%)	First-episode AP; admission within 48 h of onset; serum obtained on admission; informed consent	Not explicitly listed beyond cohort definition/consent (per Methods)	On admission (patients admitted within 48 h of onset)	SAP = POF (≥48 h) per Revised Atlanta 2012; OF by Modified Marshall (respiratory, renal, cardiovascular).	SAP (SAP) / POF vs MSAP vs MAP	Modified Marshall	≥48 h (SAP); <48 h = MSAP (transient OF)	APACHE II; Ranson (comparators)	Reported cutoffs in ROC table (APACHE II 4.0; Ranson 2.0–3.0 depending on comparison)	ICU admission and mortality reported; need for interventions listed	During index hospitalization (prospective follow-up)	ROC analyses for SAP vs MAP; SAP vs MSAP; SAP vs (MAP+MSAP).	Multivariable logistic regression: Ang-2 independently associated with SAP (e.g., OR 12.1 with cutoff 3310 pg/mL)
Espinosa_2011 [34]	2011	Spain	Case–control study (25 AP cases; 30 healthy controls)	55 (25 AP; 30 controls)	72 ± 18 years (AP cases)	36% (9/25) in AP cases	Hospitalized AP; sampling at 12 h and 5 days after admission; informed consent	Not specified beyond case/control definitions	12 h after hospitalization and day 5	Unfavorable evolution defined by complications during hospitalization: infection of pancreatic fluid collections, pancreatic necrosis, renal dysfunction, acute pulmonary insufficiency, shock, death.	Unfavorable clinical evolution vs favorable (Atlanta criteria); predicted severity (Ranson/Glasgow)	NA (Ranson/Glasgow used for predicted severity)	NA	Ranson; Glasgow (first 48 h)	Ranson ≥3 or Glasgow ≥4 (within 48 h)	Mortality reported (1 death; ICU admission for that patient)	During index hospitalization (prospective follow-up)	ROC analysis for predicting unfavorable clinical evolution; counts: unfavorable 7/25 (28%).	PDGFBB, Ang-2, HGF each AUC 0.97 for unfavorable clinical evolution
Budding_2014 [35]	2014	The Netherlands	Substudy of a randomized, double-blind, placebo-controlled trial (PROPATRIA) with prospective biomarker sampling	115 AP patients with Ang-2 samples collected within first 5 days after admission; 20 healthy controls (kidney donors)	60 ± 16 years (analysis cohort)	61.7% (female 38.3% reported)	AP with predicted severe course (APACHE II ≥8, or Imrie ≥3, or CRP >150 mg/L); symptom onset <72 h before randomization; Ang-2 sample within first 5 days after admission	ERCP-induced pancreatitis; suspected pancreato-biliary malignancy; non-pancreatic infection/sepsis from another disease; diagnosis first made at operation; immune deficiency	First 5 days after admission; median day 3 (IQR 2–4)	SAP = organ failure and/or pancreatic parenchymal necrosis on CT (Atlanta-adapted). MOF = failure of ≥2 systems same day. Infectious complications = infected necrosis, bacteremia, pneumonia, urosepsis, infected ascites (during admission and at 90-day follow-up)	SAP (SAP); multiorgan failure (MOF); infectious complications; bowel ischemia; mortality	Other (threshold-based definitions, not a scored index)	Unclear (persistence not specified; SAP includes necrosis and/or OF)	APACHE II; Imrie (comparators)	APACHE II and Imrie used as conventional predictors (thresholds per trial enrollment)	Mortality; infected pancreatic necrosis; bacteremia; pneumonia; urosepsis; infected ascites; bowel ischemia	During admission and at 90-day follow-up for infections; MOF timing analyzed for first week vs after first week	ROC analyses for SAP, MOF, infectious complications; AUCs compared with APACHE II, Imrie, CRP, LBP, PCT.	Ang-2 superior to conventional predictors for SAP/MOF/infections in this cohort.
Whitcomb_2010_US [36]	2010	United States	Prospective observational (biobank analysis)	28 AP patients (admission samples ≤3 days after pain onset) + 58	Mean 53.9 years	54%	Hospitalized with AP, recruited ≤7 days from pain onset; admission sample available	Chronic pancreatitis; missing admission serum sample; late admission >7 days	≤24 (admission sample)	Persistent OF defined as ≥48 h of any: shock (SBP<90), pulmonary insufficiency (PaO_2<60 or MV), renal insufficiency (Cr≥2 mg/dL post-rehydration or	POF (≥48 h) vs no POF; mortality reported; pancreatic necrosis noted (any time)	Other - study-specific criteria as above (not	>48h (primary endpoint).	APACHE II and Ranson (comparators)	NA (AUC comparisons; no fixed threshold)	Mortality reported; mechanical ventilation captured within pulmonary failure; pancreatic necrosis	Biomarkers at admission and daily to day 7; outcomes over index hospitalization	Admission Ang-2 predicts persistent OF; see performance metrics below	High NPV facilitates early rule-out; CRP not significant on admission

				healthy controls						dialysis, no chronic renal disease)		Marshall/SOFA).				recorded (any time). ICU admission not explicitly specified			
Whitcomb_2010_DE [36]	2010	Germany	Prospective observational (biobank analysis)	123 patients with AP + 103 healthy controls	Mean 54.0 years	67 %	Adults hospitalized for AP; recruited ≤7 days from onset of pain; admission serum collected	Chronic pancreatitis or late admission beyond 7 days	≤24 h (admission sample)	Persistent OF ≥48 h defined by same triad: shock (SBP <90), pulmonary insufficiency (PaO_2 <60 or ventilation), renal failure (Cr ≥2 mg/dL after rehydration or dialysis).	POF (≥48 h) vs no POF; mortality recorded; pancreatic necrosis noted (any time).	Other (study-defined organ failure criteria)	>48h	APACHE II (mean severe 11.9 vs mild 6.5) and Ranson (mean severe 4.4 vs mild 2.5)	NA (used descriptively, not as cut-off)	Mortality and pancreatic necrosis recorded; ICU admission not specified	Biomarkers on admission, then days 1–2, 3–4, 5–7; outcomes during hospitalization	Admission Ang-2 = 4,945 vs 2,631 pg/mL (p=0.0004); AUC 0.79 (95% CI 0.71–0.86); cut-off 2,396 pg/mL (Se=93%, Sp=63%, NPV=99%).	Ang-2 values stayed significantly higher for all measured intervals; consistent association with ongoing OF; CRP not predictive.
