# Supplementary material for: Comparative Evaluation of the Prognostic Accuracy of IL-6 and Angiopoietin-2 for Early Severity Assessment in Acute Pancreatitis: A Systematic Review
Source: Diseases. 2026 Jan 7;14(1):24. doi: 10.3390/diseases14010024 (PMC12839801; doi:10.3390/diseases14010024)
Supplement: Supplementary file 1 [file diseases-14-00024-s001.zip › Table S4.pdf]

Table S4. Biomarker measurement characteristics and diagnostic performance in included studies.

№	Study_ID	Biomarker (IL6/ANG2)	Sampling_Window_h	Sampling_Anchor (Onset/Admission)	Assay_Method (ELISA/CLIA/Other)	Manufacturer_Kit	Units (pg/mL)	Cutoff_Value	Sensitivity	Specificity	AUC	AUC_95CI_Lower	AUC_95CI_Upper	Notes_Biomarker
1	Sathyanarayan_2007 [23]	IL-6	≤72	Onset	ELISA	Diaclone (France)	pg/mL	122	81.8	77.7	0.823	NA	NA	IL-6 (day 3) associated with organ failure; TNF-α and IL-10 non-significant; mortality 10%
2	Kolber_2018 [24]	IL-6	≤ 24	Admission and Day 2	ECLIA (Electrochemiluminescence Immunoassay)	Cobas 8000 Analyzer, Roche Diagnostics (Mannheim, Germany)	pg/mL	211 (pg/mL for SAP), 262 (pg/mL for vital organ failure), 229 (pg/mL for ICU transfer/death)	57 (for SAP @211 pg/mL); 62 (for OF @262 pg/mL)	82 (for SAP); 88 (for OF)	0.753 (SAP); 0.767 (OF); 0.781 (ICU/death)	0.590 (SAP)	0.917 (SAP)	IL-6 positively correlated with Ang-2 (r = 0.26 on day 1; r = 0.54 on day 2, p = 0.004); predicted SAP and organ failure with AUC ≈ 0.75–0.78; correlated with PCT, CRP, and renal injury markers (KIM-1, L-FABP).
3	Li_2022 [25]	IL-6	≤ 48	Admission (within 48 h of onset)	ELISA	Double-antibody sandwich ELISA (Hospital Inspection Center)	pg/mL	121.10 (SAP); 54.16 (organ failure); 219.30 (IPN)	67.65 % (for SAP @121.1 pg/mL)	67.74 % (for SAP @121.1 pg/mL)	0.69 (SAP); 0.72 (OF); 0.81 (IPN); 0.75 (Mortality)	0.56 (for SAP)	0.82 (for SAP)	IL-6 and CRP both correlated with severity; IL-6 outperformed CRP for IPN and mortality prediction but not for SAP/OF.
4	Yao_2024 [26]	IL-6	≤ 48	Within 48 h of symptom onset (admission testing)	Multiplex microsphere flow immunofluorescence	12-cytokine detection kit (Qingdao Ruisikai Biotechnology Co., Ltd.); analyzer: Beckman Navios flow cytometer	pg/mL	24.67 (for SAP)	87.23 % (for SAP @24.67 pg/mL)	66.92 % (for SAP @24.67 pg/mL)	0.7875 (for SAP)	0.7280	0.8470	AUCs also reported for cytokine associations with ANC, APFC, pleural effusion, ascites via regression models.
5	Wu_2025 [27]	IL-6	≤ 6 (from admission)	Admission	Not reported	Not reported	pg/mL	27.4	87%	73%	0.86	Not reported	Not reported	Ultra-early indicators tested within 6 h; IL-6 identified as an independent risk factor; combination model (IL-6 + D-dimer + calcium) AUC 0.88.
6	Jain_2018 [28]	IL-6	≤ 72 (cytokine cohort measured at day 3 of onset)	Day 3 from onset (patients who presented within 72 h)	ELISA	R&D Systems (Minneapolis, MN, USA)	pg/mL	160	86% (for severe AP at IL-6 >160 pg/mL, day 3)	82% (for severe AP at IL-6 >160 pg/mL, day 3)	0.83	0.71	0.95	Combination “SIRS at admission + IL-6 >160 pg/mL (day 3)” yielded sensitivity 79%, specificity 95%, PPV 85%, NPV 93%.
7	Bhowmick_2024 [29]	IL-6	Blood drawn within 24 h of admission	Admission (patients presented within 48 h of onset)	ELISA	NA (not specified)	pg/mL	46.379 pg/mL (>)	96.15% (95% CI 80.4–99.9%)	95.83% (95% CI 78.9–99.9%)	0.990	0.911	1.000	IL-6 range 11.92–373.16 pg/mL; mean 82.13 pg/mL; median 57.4 pg/mL; ROC-derived cutoff predicts SAP with high accuracy
8	Sternby_2017 [30]	IL-6	Within first 24–36 h (early course)	Admission	ELISA	Not reported	pg/mL	**>50 pg/mL** (по ROC mild vs non-mild)	86%	46%	0.72	NA	NA	IL-6 higher in non-mild AP; ROC performed for mild vs non-mild; cutoff 50 pg/mL; moderate predictive strength.
9	Dumnicka_2017 [31]	Ang-2	24 h and 48 h from onset (on admission and Day 2)	Admission within 24 h from symptom onset; Day 2 at 48 h	ELISA	Quantikine ELISA Human Angiopoietin-2 (R&D Systems, USA)	pg/mL	5.92 ng/mL	100% (SAP, 24 h)	92% (SAP, 24 h)	0.946 (SAP, admission ≤24 h)	NA	NA	Ang-2 correlated with D-dimer and ISTH DIC score; highest diagnostic utility for SAP at admission.
10	Huang_2020 [32]	Ang-2	Blood collected on admission within 24 h after onset (single time point)	Admission (<24 h from onset)	ELISA	R&D Systems (Human Ang-2 ELISA	pg/mL	11.49 μg/L	93.8% (for SGI)	82.6% (for SGI)	0.916 (for SGI)	NA	NA	Additional ROC results: MODS AUC 0.980 (cutoff 11.76 μg/L; Se 100%; Sp 95.2%); pancreatic necrosis AUC 0.905 (cutoff 11.90 μg/L; Se 91.7%; Sp 80.8%); ICU admission AUC 0.903 (cutoff 8.945 μg/L; Se 77.5%; Sp 88.9%); feeding intolerance AUC 0.940 (cutoff 9.81 μg/L; Se 100%; Sp 79.7%); mortality AUC 0.924 (cutoff 15.31 μg/L; Se 100%; Sp 87.3%).

11	Zhang_2016 [33]	Ang-2	On admission (patients admitted within 48 h of onset)	Admission	ELISA	Quantikine Human Angiopoietin-2 ELISA (R&D Systems)	pg/mL	For SAP vs (MAP+MSAP): 3310.0 pg/mL; for SAP vs MAP: 3015.0 pg/mL; for SAP vs MSAP: 3310.0 pg/mL.	SAP vs (MAP+MSAP): 75.68%; SAP vs MAP: 75.68%; SAP vs MSAP: 75.68%.	SAP vs (MAP+MSAP): 79.52%; SAP vs MAP: 90.00%; SAP vs MSAP: 69.77%.	SAP vs (MAP+MSAP): 0.808 (95% CI 0.726–0.874); SAP vs MAP: 0.878 (0.784–0.942); SAP vs MSAP: 0.743 (0.633–0.834)	0.726 (SAP vs [MAP+MSAP]); 0.784 (SAP vs MAP); 0.633 (SAP vs MSAP).	0.874 (SAP vs [MAP+MSAP]); 0.942 (SAP vs MAP); 0.834 (SAP vs MSAP).	Multivariable logistic regression: Ang-2 independently predicted SAP (e.g., OR 12.1 with cutoff 3310 pg/mL for all AP patients)
12	Espinosa_2011 [34]	Ang-2	12 h after hospitalization and day 5	Admission (post-admission 12 h)	ELISA	R&D Systems (ELISA products)	ng/mL (as reported)	10 ng/mL (for unfavorable clinical evolution)	100% (for unfavorable clinical evolution)	88% (for unfavorable clinical evolution)	0.97 (for unfavorable clinical evolution)	NA	NA	Ang-2 higher in unfavorable vs favorable evolution groups at 12 h; also elevated vs controls.
13	Buddingh_2014 [35]	Ang-2	First 5 days after admission; median day 3 (IQR 2–4)	Admission (early sampling window); symptom onset <72 h before randomization	ELISA	R&D Systems (Human Ang-2 ELISA)	mg/L (as reported)	For SAP: 4.56 mg/L; for MOF: 5.01 mg/L; for infectious complications: 4.51 mg/L (optimal by ROC)	SAP 81.1%; MOF 72.2%; infections 79.5%	SAP 76.9%; MOF 73.2%; infections 76.3%	SAP 0.851; MOF 0.784; infections 0.816; bowel ischemia 0.895; mortality 0.865	NA	NA	Median Ang-2 higher with MOF (7.4 vs 4.0 mg/L), infections (6.4 vs 3.2 mg/L), bowel ischemia (11.9 vs 4.0 mg/L), and non-survivors (11.9 vs 4.0 mg/L); all p<0.001 (except MOF after first week p=0.049).
14	Whitcomb_2010_US [36]	Ang-2	≤24 (admission sample)	**Admission** (≤3 days from pain onset for this subset)	ELISA (Luminex Bio-Plex platform)	R&D Systems; Ang-2 antibodies BAM0981 (Det) & MAB098 (Cap)	pg/mL	1,910 pg/mL	83%	91%	0.94 (95% CI 0.76–0.99)	0.76	0.99	Elevated through day 7 among severe; CRP not significant at admission
15	Whitcomb_2010_DE [36]	Ang-2	≤24 h (admission sample)	Admission	ELISA	R&D Systems (same lot as UPMC)	pg/Ml	2,396 pg/mL	93%	63%	0.79 (95% CI 0.71–0.86)	NA	NA	Ang-2 outperformed CRP; similar diagnostic accuracy to APACHE II; NPV 99%; values elevated through day 7.
