# Supplementary material for: Comparative Evaluation of the Prognostic Accuracy of IL-6 and Angiopoietin-2 for Early Severity Assessment in Acute Pancreatitis: A Systematic Review
Source: Diseases. 2026 Jan 7;14(1):24. doi: 10.3390/diseases14010024 (PMC12839801; doi:10.3390/diseases14010024)
Supplement: Supplementary file 1 [file diseases-14-00024-s001.zip › Table S5.pdf]

Table S5. Summary of Key Findings for Interleukin-6 and Angiopoietin-2 in Acute Pancreatitis

**Feature**	**Interleukin-6 (IL-6)**	**Angiopoietin-2 (Ang-2)**
Pathophysiological pathway	Reflects systemic inflammatory activation and the cytokine-mediated response associated with SIRS.	Reflects endothelial dysfunction, increased vascular permeability, and capillary leak syndrome.
Temporal profile / peak	Typically peaks within the first 24 hours after symptom onset and declines rapidly thereafter.	Exhibits sustained elevation from admission through 48-72 hours and beyond, particularly in severe disease.
Prognostic accuracy (AUC)	Ranges from 0.69 to 0.99 across studies, with substantial heterogeneity related to sampling time, disease phase, and study design.	Demonstrates consistently high AUC values (0.85-0.98), showing lower variability across different study designs and sampling windows.
Predictive outcomes	Associated with severe acute pancreatitis, early organ dysfunction, and adverse clinical outcomes, with indirect associations with infected necrosis and mortality.	Predicts persistent organ failure (POF), multiple organ dysfunction syndrome (MODS), infected necrosis, and mortality.
Diagnostic stability	Highly sensitive to sampling time; diagnostic performance varies depending on the phase of disease.	Shows greater stability and consistency across different study designs and timing of measurement.
Clinical role	May be considered an ultra-early marker of systemic inflammatory burden during the initial hours of hospital admission.	May serve as an indicator of endothelial injury and sustained risk of severe complications and organ failure.
